# Severe MRSA Enterocolitis Caused by a Strain Harboring Enterotoxins D, G, and I

**DOI:** 10.3201/eid2305.161644

**Published:** 2017-05

**Authors:** Marco Bergevin, Alain Marion, David Farber, George R. Golding, Simon Lévesque

**Affiliations:** Cité de la Santé de Laval, Laval, Québec, Canada (M. Bergevin, A. Marion, D. Farber);; National Microbiology Laboratory, Public Health Agency of Canada, Winnipeg, Manitoba, Canada (G.R. Golding);; Laboratoire de Santé Publique du Québec, Institut National de Santé Publique du Québec, Sainte-Anne-de-Bellevue, Québec, Canada (S. Lévesque)

**Keywords:** MRSA, enterocolitis, enterotoxins, methicillin-resistant *S. aureus*, *Staphylococcus aureus*, bacteria, bacterial infection, drug resistance, staphylococci, Canada

## Abstract

We describe a case of methicillin-resistant *Staphylococcus aureus* (MRSA) enterocolitis in a healthy adult with previous antibiotic exposure. Colonoscopy revealed diffuse colitis and mild ileitis without ulceration. Stool cultures demonstrated abundant growth of MRSA and absent normal flora. Oral vancomycin treatment was effective and seems to be the consensus choice for therapy.

*Staphylococcus aureus* was recognized as a cause of antibiotic-associated colitis (AAC) in the mid-20th century (*1*,*2*). *Clostridium difficile* was later identified as the primary cause of AAC, and appreciation of *S. aureus* as a potential etiology declined (*2*). Methicillin-resistant *S. aureus* (MRSA) has also been implicated as a cause of AAC, with most reports coming from Japan. We report a case of MRSA enterocolitis in Canada caused by a strain harboring multiple enterotoxins.

In 2014, a 22-year-old woman sought care after 10 days of acute and profuse diarrhea, abdominal cramping, nausea, and weight loss of 5 lbs. She had 10–30 bowel movements a day and had observed blood-tainted stool. The patient reported a history of migraine and depression but was otherwise healthy. She worked in a pet store and had not been hospitalized. In the previous 2 months, she had been treated for chlamydia with a single course of azithromycin and cefixime. Subsequently, she received a 10-day course of azithromycin followed by 10 days of moxifloxacin for bronchopneumonia. Her family history revealed Crohn’s colitis in her father.

The patient was afebrile; blood pressure was 104/58 mm Hg and pulse 91 bpm. Her abdomen was soft without rebound tenderness. Laboratory test results revealed a normal leukocyte count but a C-reactive protein level of 76 mg/L. Her initial diagnosis was with bacterial enteritis, and she was sent home with an order for stool cultures and oral ciprofloxacin to be started after stool collection. On follow-up 3 days later, she felt better but was still having 6 bowel movements a day and bloody stool. She remained afebrile, but her blood pressure was 86/57 mm Hg and pulse 104 bpm. Colonoscopy with ileoscopy was performed to explore the possibility of inflammatory bowel disease; results revealed diffuse acute colitis with an inhomogeneous pattern (Figure, panels A–D) and minimal focal erythema in the terminal ileum. *C. difficile* testing was negative for glutamate dehydrogenase antigen and toxins by Quik-Chek Complete (Alere, San Diego, CA, USA). Testing of the 3 stool cultures demonstrated absent normal flora and abundant growth of MRSA. Eight days after initial consultation, she was seen by an infectious disease specialist and prescribed oral vancomycin (125 mg 4×/d for 10 d). She had an excellent and rapid clinical response to treatment, and her symptoms did not recur. The patient was found to have nasal colonization with MRSA.

**Figure:**
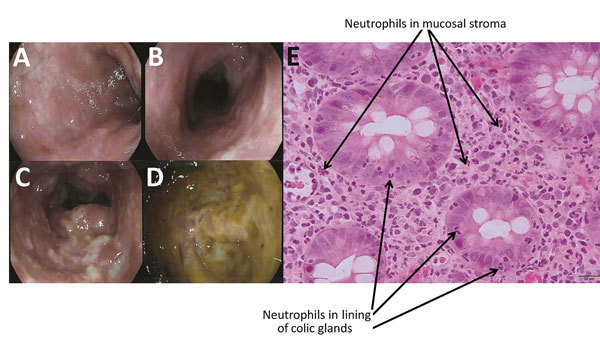
Endoscopic imagery of the distal sigmoid colon (A), proximal sigmoid colon (B), descending colon (C), and base of cecum (D), revealing diffuse colitis with mucosal erythema, edema, and mucopurulent exudate without ulceration. Colonic biopsy (E) demonstrating neutrophilic infiltrates (indicated with arrows) in the epithelial lining of the colic glands and the mucosal stroma compatible with mild active colitis without signs of chronicity.

Colonic biopsy confirmed colitis without chronicity (Figure, panel E), and ileal biopsy revealed focal active ileitis. Because MRSA colitis is rare, the strain was sent to the Laboratoire de Santé Publique du Québec for identification and further characterization (Technical Appendix). Genetic typing was positive for *nuc* and *mecA* genes, confirming MRSA. The strain was negative for Panton–Valentine leukocidin, toxic shock syndrome toxin 1, enterotoxins A, B, C, E, and H, and exfoliative toxins A and B, but it harbored genes for enterotoxins D, G, and I. The *spa* type was t067, corresponding to the epidemic type in Canada, CMRSA2 (also known as USA100/800, New York, sequence type 5). This strain was resistant to levofloxacin, erythromycin, and clindamycin but susceptible to all remaining antibiotics (online Technical Appendix).

*S. aureus* enterocolitis has been recognized as a cause of AAC since 1948, and MRSA colitis is a variant of this disease. Most cases were reported from Japan (*1*); the validity of these reports has been questioned because of insufficient effort to rule out *C. difficile*, but recent reviews support the existence of the syndrome (*3*). In North America, few cases of MRSA colitis have been identified; however, this cause of enteritis might be underreported because few physicians order stool cultures for hospitalized patients with AAC (*1*,*4*). Cases of staphylococcal enteritis might occur when dysbiosis permits *Staphylococcus* overgrowth and toxin secretion causes bowel inflammation (*1*). The multiple antibiotics that had been taken by this patient presumably induced dysbiosis, MRSA overgrowth, and toxin-mediated enterocolitis. The relative contribution of the different toxins to the syndrome are unknown. Gut colonization is believed to originate from the upper respiratory tract of colonized persons (*5*).

In previous reported cases of MRSA enterocolitis, few authors reported the presence of *Staphylococcus* toxins, mainly enterotoxin A. Although enterotoxin A is the most common toxin in *Staphylococcus*-related food poisoning, the enterotoxin D we found is reported to be the second most common, supporting its role in this patient’s enterocolitis (*6*). Enterotoxins G and I are not as well-studied but were associated with a food poisoning outbreak in Taiwan (*7*). Staphylococcal enterotoxins G and I induce enterocolitis by a combination of direct enterocyte cytopathy mediated by epidermal cell differentiation inhibitor toxins (disrupting the epithelial barrier) and enterotoxin superantigen-induced mucosal T-cell activation (*8*).

The risk factors for *S. aureus* enterocolitis include age, hospitalization, abdominal surgery, immunosuppression, gastric acid suppression, MRSA colonization, and previous antibiotic therapy. Fluoroquinolone use seems to be particularly associated with this complication (*1*). MRSA enterocolitis might be an underrecognized cause of AAC because stool culture in patients hospitalized for >72 hours has been discouraged (*9*). MRSA enterocolitis should be considered once *C. difficile* colitis and *Klebsiella oxytoca* colitis have been ruled out (*10*). Physicians should consider stool cultures for severe or prolonged AAC, and microbiology laboratories should report *S. aureus* overgrowth in stool. Oral vancomycin was effective in this case and seems to be the consensus choice for therapy based on previous reports.

Technical Appendix Methods for identification and further characterization of isolated MRSA strain.
