# Supplementary material for: Severe MRSA Enterocolitis Caused by a Strain Harboring Enterotoxins D, G, and I
Source: Emerg Infect Dis. 2017 May;23(5):865–7. doi: 10.3201/eid2305.161644 (PMC5403038; doi:10.3201/eid2305.161644)
Supplement: Technical Appendix — Methods for identification and further characterization of isolated MRSA strain. [file 16-1644-Techapp-s1.pdf]

Article DOI: http://dx.doi.org/10.3201/eid2305.161644

Severe MRSA Enterocolitis Caused by a Strain Harboring Enterotoxins D, G, and I

Technical Appendix

Methods

At LSPQ, confirmation of *S. aureus* identification was performed by PCR amplification of the *nuc* gene (*1*) and confirmation of methicillin resistance by PCR amplification of the *mecA* gene (*2*). The detection of the *lukS-PV* and *lukF-PV* genes coding for PVL toxin and the toxic shock syndrome toxin 1 (TSST-1) were done by PCR (*3*,*4*). Molecular characterization was performed using *spa* typing (*5*). The obtained genotype was associated with its corresponding MRSA epidemic type according to the standard protocol of the National Microbiology Laboratory (NML) (*6*). The strain was sent to the NML for further toxin characterization. The detection of *Staphylococcus* enterotoxin A, B, C, D, E, G, H, and I, as well as exfoliative toxin A and B were done by PCR (*4*,*7*).

The minimum inhibitory concentrations (MICs) were determined by broth microdilution according to CLSI guidelines (*8*–*10*). The following antibiotics were tested at concentrations varying from 0.06 to 64 mg/l (except for rifampin: 0.016–16 mg/l): daptomycin, doxycycline, fusidic acid, levofloxacin, linezolid, rifampin, trimethoprim-sulfamethoxazole and vancomycin. Susceptibility to clindamycin and to erythromycin were determined by disk diffusion and inducible resistance to clindamycin was detected by D-test according to CLSI guidelines (*8*). High-level mupirocin resistance was determined by disk diffusion according to CLSI guidelines (*8*). MICs were interpreted using breakpoints established by CLSI, except for fusidic acid where EUCAST breakpoints were used (*11*).

References

1. Tawil N, Mouawad F, Lévesque S, Sacher E, Mandeville R, Meunier M. The differential detection of methicillin-resistant, methicillin-susceptible and borderline oxacillin-resistant *Staphylococcus aureus* by surface plasmon resonance. Biosens Bioelectron. 2013;49:334–40. PubMed http://dx.doi.org/10.1016/j.bios.2013.05.031

2. Geha DJ, Uhl JR, Gustaferro CA, Persing DH. Multiplex PCR for identification of methicillin-resistant staphylococci in the clinical laboratory. J Clin Microbiol. 1994;32:1768–72. PubMed

3. Lina G, Piémont Y, Godail-Gamot F, Bes M, Peter MO, Gauduchon V, et al. Involvement of Panton-Valentine leukocidin-producing *Staphylococcus aureus* in primary skin infections and pneumonia. Clin Infect Dis. 1999;29:1128–32. PubMed http://dx.doi.org/10.1086/313461

4. Becker K, Roth R, Peters G. Rapid and specific detection of toxigenic *Staphylococcus aureus*: use of two multiplex PCR enzyme immunoassays for amplification and hybridization of staphylococcal enterotoxin genes, exfoliative toxin genes, and toxic shock syndrome toxin 1 gene. J Clin Microbiol. 1998;36:2548–53. PubMed

5. Harmsen D, Claus H, Witte W, Rothgänger J, Claus H, Turnwald D, et al. Typing of methicillin-resistant *Staphylococcus aureus* in a university hospital setting by using novel software for spa repeat determination and database management. J Clin Microbiol. 2003;41:5442–8. PubMed http://dx.doi.org/10.1128/JCM.41.12.5442-5448.2003

6. Golding GR, Campbell JL, Spreitzer DJ, Veyhl J, Surynicz K, Simor A, et al.; Canadian Nosocomial Infection Surveillance Program. A preliminary guideline for the assignment of methicillin-resistant *Staphylococcus aureus* to a Canadian pulsed-field gel electrophoresis epidemic type using spa typing. Can J Infect Dis Med Microbiol. 2008;19:273–81. PubMed http://dx.doi.org/10.1155/2008/754249

7. Jarraud S, Cozon G, Vandenesch F, Bes M, Etienne J, Lina G. Involvement of enterotoxins G and I in staphylococcal toxic shock syndrome and staphylococcal scarlet fever. J Clin Microbiol. 1999;37:2446–9. PubMed

8. Clinical Laboratory Standards Institute. Performance standards for antimicrobial susceptibility testing. 26th ed. CLSI supplement M100S. Wayne (PA): The Institute; 2016.

9. Clinical Laboratory Standards Institute. Methods for dilution antimicrobial susceptibility tests for bacteria that grow aerobically; approved standard. 10th edition. Vols. M07–A10. Wayne (PA): The Institute; 2015.

10. Clinical Laboratory Standards Institute. Performance standards for antimicrobial disk susceptibility test; approved standard. 12th edition. Vols. M02–A12. Wayne (PA): The Institute; 2015.

11. European Committee on Antimicrobial Susceptibility Testing. Clinical breakpoints: breakpoint table for bacteria [cited 2016 Jan 1]. http://www.eucast.org/fileadmin/src/media/PDFs/EUCAST_files/Breakpoint_tables/v_6.0_Breakpoint_table.pdf
